# A comparison of ordinal regression models in an analysis of factors associated with periodontal disease

**DOI:** 10.4103/0972-124X.75909

**Published:** 2010

**Authors:** Shivalingappa B. Javali, Parameshwar V. Pandit

**Affiliations:** *Department of Biostatistics, SDM College of Dental Sciences, Dharwad, India*; 1 *Department of Statistics, Bangalore University, Bangalore, Karnataka, India*

**Keywords:** CPITN, ordinal data and built-in link functions, ordinal regression model, periodontal disease

## Abstract

**Aim:**

The study aimed to determine the factors associated with periodontal disease (different levels of severity) by using different regression models for ordinal data.

**Design:**

A cross-sectional design was employed using clinical examination and ‘questionnaire with interview’ method.

**Materials and Methods:**

The study was conducted during June 2008 to October 2008 in Dharwad, Karnataka, India. It involved a systematic random sample of 1760 individuals aged 18-40 years. The periodontal disease examination was conducted by using Community Periodontal Index for Treatment Needs (CPITN).

**Statistical Analysis Used:**

Regression models for ordinal data with different built-in link functions were used in determination of factors associated with periodontal disease.

**Results:**

The study findings indicated that, the ordinal regression models with four built-in link functions (logit, probit, Clog-log and nlog-log) displayed similar results with negligible differences in significant factors associated with periodontal disease. The factors such as religion, caste, sources of drinking water, Timings for sweet consumption, Timings for cleaning or brushing the teeth and materials used for brushing teeth were significantly associated with periodontal disease in all ordinal models.

**Conclusions:**

The ordinal regression model with Clog-log is a better fit in determination of significant factors associated with periodontal disease as compared to models with logit, probit and nlog-log built-in link functions. The factors such as caste and time for sweet consumption are negatively associated with periodontal disease. But religion, sources of drinking water, Timings for cleaning or brushing the teeth and materials used for brushing teeth are significantly and positively associated with periodontal disease.

## INTRODUCTION

Periodontal disease is the major component of oral health that is often measured in epidemiologic studies on an ordinal scale. But data of this type are generally reduced for analysis to a dichotomy. Several statistical models have been developed to make use of information in ordinal response data, but those techniques have not been much used in analyzing data corresponding to epidemiologic studies. In this article, we discuss an overview of logistic regression models for ordinal data based on cumulative and conditional probabilities. The most popular ordinal regression models are embedded under different link functions in the framework of generalized linear models. The application of the proposed model with different link functions to data of periodontal disease of 1,760 random samples confirmed that generalized linear models are easy to use and interpret but gave results quite different to those obtained using binary (simple) logistic regression after dichotomizing outcome in the conventional way.

Many variants of regression models for analyzing ordinal response variables have been developed and described during the past years.[1–20] Compared to frequently used methods for binary and nominal data, ordinal regression models have the advantage that they make full use of ranked data.[13,16,18] Nevertheless, these models have been underutilized in biomedical and epidemiological research. Therefore, epidemiological data analyses concerning risk factors rely heavily on regression models. The choice of a model is largely determined by the scale of measurement of the response variable.[3]

Epidemiologists and statisticians are often interested in estimating the risk of adverse events, originally measured on interval scale (such as attachment loss), but they often choose to decide the outcome on two or more categories in order to compute an estimate of effects of covariates. Similarly, some response variable originally measured on an ordinal scale (severity of periodontal disease) is often categorized into several binary variables during statistical analysis. As a motivating example, the Community Periodontal Index for Treatment Needs (CPITN) was used to assess the pattern or severity of periodontal disease. The severity of periodontal disease response was recorded on a 5-level ordinal scale. Usually such data are analyzed by ordinal logistic model rather than by creating dichotomy among the levels of periodontal disease (with and without periodontal disease).

Although such approaches are not incorrect, they often result in loss of information due to collapsing of some groups of the response variable and considerable amount of loss of statistical power in results. Therefore, if researchers wish to study the effects of independent variables on all levels of ordered categorical response, an ordinal regression method must be appropriately chosen in order to obtain valid results. But in statistical literature, several statistical models for ordinal response have been proposed; however, their utilization in the dental epidemiological and biomedical literature has been minimal and least. Evaluation of the usefulness of ordinal models in dental epidemiological research with particular emphasis on model formation includes severity of periodontal disease as a response variable.

In this study, the ordinal regression model was used to model relationship between the ordinal outcome (i.e., different levels of severity of periodontal disease) and independent variables. The framework of ordinal regression model is described with data set in the following section.

### Application - CPITN index data

Let Y (periodontal disease) be a categorical response variable with k+1 (k=4) ordered categories coded as 0, 1, 2, 3, 4. Here, we consider the severity of periodontal disease as a response variable given by ordered categories, with higher values indicating more severity, as given below:

$$Y(CPITN) if=\{\begin{array}{l} \text{0, if No periodontal disease (CPITN=0)}\\ \text{1, if Bleeding on gentle probing (gums) (CPITN=1)}\\ \text{2, if Calculus felt during the probing (CPITN=2)}\\ \text{3, if Periodontal pocket depth between 3.5 to 5.5 mm (CPITN=3)}\\ \text{4, if Periodontal pocket depth 6 mm or more (CPITN=4)} \end{array}$$

The major goal of this article was to use applications of an ordinal logistic regression model for modeling CPITN with different built-in link functions[20] to predict the probability of occurrence of periodontal disease. The following built-in link functions were considered.

1. Logit link function = *f*{*π*}=log$\left\{\frac{\pi }{1-\pi }\right\}$=*α*+*βx*

2. Probit link function = *f*{*π*} = *φ*^−1^(*π*)

3. Clog-log link function = *f*{*π*} = log{− log(1−*π*(*x*))} = *α* + *βx*

4. nlog-log link function = *f*{*π*} = − log(log *π*) = *α* + *βx*

The strengths of the ordinal regression model with above four built-in link functions are briefly described. Firstly, many indicators concerning periodontal disease outcome (CPITN) are frequently measured on an ordinal scale. Thus, the ordinal regression model seems to have a broad marketplace to analyze diverse periodontal disease outcomes. Second, comparable to logistic regression model, an ordinal regression model can be used to perform the following tasks:

1. To identify significant independent variables that influence the ordinal response, i.e., periodontal disease
2. To describe the direction of the relationship between the ordinal outcome, i.e., periodontal disease, and the independent variables
3. To analyze for all levels of the ordinal outcome, i.e., periodontal disease, and subsequently evaluate and predict validity of the regression model.

Third, the four different link functions are used to model the effects of independent variables on the ordinal response. Finally, the model assumes that the relationship between the ordinal outcome and the independent variable is independent of the category. This assumption implies that the corresponding regression coefficients in the link function are equal for each cut-off point.[21] Therefore, it is easy to construct and interpret the ordinal regression model, which requires only one model assumption and produces only one set of regression coefficients.

## MATERIALS AND METHODS

### Study area

The cross-sectional study was conducted during June to October 2008 in Dharwad, Karnataka, India. Dharwad is situated in north Karnataka and is one of the educational centers.

### Study population and sampling procedure

The cross-sectional study involved a systematic random sample of 1760 individuals aged 18-40 years. Sample size was determined based on the results of pilot study, which showed that standard deviation (SD) of CPITN score was 0.8120 under precision of 5% and confidence level of 99%. The sample size was estimated to be 1,756 ≅ 1,760. The mean age of the study subjects was 34.26±7.28 years.

### Clinical examination

The periodontal disease (CPITN) examination was carried out by two qualified dental surgeons using the standardized and widely accepted procedure recommended by the WHO report on oral health,[22] with mouth mirror, CPITN probe, dental explorer, disposable gloves and sterilized instruments under artificial light. Before the start of the actual study, a pilot study was conducted to assess the intra- and inter-examiner agreement for recording CPITN scores on a convenient sample size of 140 study subjects. The intra-examiner agreement was 0.8719 (first examiner) and 0.7193 (second examiner), respectively. The inter-examiner (between the two examiners) agreement was found to be 0.8795.

Besides the data on periodontal disease (CPITN), the data were also collected on various characteristics, like socioeconomic–socio-demographic characteristics, food habits, eating habits, oral hygiene practices and deleterious habits, using ‘structured questionnaire and personal interview’ procedure. The CPITN (periodontal disease) data was considered as an ordinal response variable. The 17 independent variables of the study were as follows: Socioeconomic–socio-demographic characteristics included gender (male=1, female=0), age (as a continuous variable), religion (Hindu=1, non-Hindu=0), caste (SC/ST/OBC=0, GM=1), Socio-Economic status (low=0, intermediate=1, high=2)[23] and family size (as continuous variable). Food habits included types of diet (vegetarian=0, non-vegetarian=1). Eating habits were assessed in terms of frequency of sweet consumption (per day) (once=1, twice=2, more than twice=3). Oral hygiene practices were measured in terms of oral hygiene habits (finger=0, brush/others=1), frequency of brushing (once=1, twice or more=2), methods of brushing (circular/vertical=1, horizontal=2), materials used for brushing teeth (paste/powder=1, others=2), types of toothpaste (non-fluoridated=0, fluoridated=1), duration of change of toothbrush (1-3 months=0, >3 months=1) and mouth rinsing habit (no=0, yes=1). The deleterious habits were assessed through smoking habit (no=0, yes=1) and chewing habit (no=0, yes=1). Since a cross-sectional design was adapted for the present study, data collection regarding the above-mentioned characteristics was based on the information at the time of data collection and not on past history.

### Data analysis

The major goal of this article was to utilize the application of ordinal logistic regression model with different built-in link functions, viz., logit, probit, Clog-log and nlog-log, in the estimation of significant factors associated with periodontal disease. There is no clear-cut method to determine the order of preference of using different link functions. However, the logit link and Clog-log link are generally suitable for analyzing the ordered categorical data evenly distributed among all categories. Lastly, the investigators were also interested in establishing the fitting performance of ordinal regression model with different built-in link functions, viz., logit, probit, Clog-log and nlog-log, ordinal response by using log likelihood and Akaike information criteria (AIC). Statistical significance was set at 5% level of significance (*P*<.05)[24–25]

## RESULTS

The periodontal disease Community Index for Treatment needs (CPITN) ordinal data set was analyzed. Comparisons in terms of estimates, log likelihood and AIC values in particular were carried out for model with four built-in link functions and these are discussed and presented in this article. The results of estimates of ordered regression model with four built-in link functions on five categories of periodontal disease are presented in Table 1.

**Table 1:** Estimates of determinants of five categories of periodontal disease using ordinal regression model with different built-in link functions

Variables	Ordinal regression model with							
	Logit link function		Probit link function		Clog-log link function		nlog-log link function	
	Estimate	SE	Estimate	SE	Estimate	SE	Estimate	SE
Gender	−0.103	0.098	−0.045	0.057	−0.079	0.059	−0.079	0.059
Age (in years)	0.019	0.032	0.010	0.019	0.019	0.020	0.019	0.020
Religion	0.266*	0.093	0.159*	0.054	0.121*	0.057	0.121*	0.057
Caste	−0.158*	0.054	−0.088*	0.031	−0.059*	0.033	−0.059*	0.033
Socioeconomic status	−0.090	0.069	−0.048	0.040	−0.046	0.042	−0.046	0.042
Family size	0.030	0.128	0.028	0.075	0.020	0.078	0.020	0.078
Staple food	0.074	0.155	0.040	0.089	−0.073	0.093	−0.073	0.093
Sources of drinking water	0.633*	0.161	0.343*	0.094	0.373*	0.099	0.373*	0.099
Dietary habits	0.001	0.089	0.004	0.052	−0.023	0.054	−0.023	0.054
Time for sweet consumption	−0.655*	0.264	−0.398*	0.158	−0.488*	0.163	−0.488*	0.163
Frequency of sweet consumption	0.241	0.307	0.172	0.182	0.201	0.190	0.201	0.190
Oral hygiene habits	0.024	0.087	0.014	0.051	0.042	0.053	0.042	0.053
Frequency of brushing	0.036	0.122	0.019	0.071	0.043	0.074	0.043	0.074
Timings of cleaning teeth	0.590*	0.114	0.367*	0.069	0.481*	0.071	0.481*	0.071
Methods of brushing	−0.159	0.097	−0.097	0.056	−0.065	0.058	−0.065	0.058
Materials used for brushing teeth	0.343*	0.140	0.174*	0.082	0.167*	0.087	0.167*	0.087
Type of toothpaste	−0.118	0.112	−0.067	0.066	−0.007	0.069	−0.007	0.069
Mouth-rinsing habit	0.168	0.097	0.093	0.057	0.056	0.060	0.056	0.060
Smoking habit	0.066	0.109	0.025	0.064	0.030	0.067	0.030	0.067
Chewing habit	0.182	0.178	0.141	0.104	0.157	0.108	0.157	0.108
Alcohol habit	0.270	0.180	0.176	0.105	0.204	0.109	0.204	0.109
Threshold (category 1)	0.189	0.708	0.067	0.409	−0.728	0.432	−0.728	0.432
Threshold (category 2)	1.374	0.708	0.745	0.409	0.312	0.429	0.312	0.429
Threshold (category 3)	2.606	0.710	1.505	0.409	1.219	0.429	1.219	0.429
Threshold (category 4)	4.219	0.715	2.441	0.411	2.083	0.430	2.083	0.430

* Significant at 5% level of significance (*P*<.05); SE = Standard error

It shows that, three thresholds of the model equation are significantly different from zero and substantially contributed to the values of the response probability in different categories in regression model with four built-in link functions. Out of 21 covariates, only 6 covariates are significantly associated with periodontal disease, in which caste and time for sweet consumption exhibited negative regression coefficients, indicating that these are negatively associated with CPITN. This means that, they are likely to decrease the higher-order scores of CPITN. However, the four covariates, viz., religion, sources of drinking water, timings of cleaning teeth and materials used for brushing teeth, are positively associated with CPITN. These significant covariates exhibited positive regression coefficients. This indicates that, these are likely to increase with the higher-order scores of CPITN in all four built-in link functions.

Further, according to order of suitability, the ordinal regression model with Clog-log built-link function is a better fit (−1908.49) as compared to nlog-log built-in link function (−1992.05), logit built-in function (−2078.36) and probit built-in link function (−2099.90). This is also supported by AIC vales. AIC is smallest in ordinal regression model with Clog-log built-in link function (2.19), followed by nlog-log built-in link function (2.29), logit built-in link function (2.39) and probit built-in link function (2.41) [Table 2]. Therefore, we conclude that the ordinal regression model with Clog-log built-in link function is a better fit as compared to model with logit, nlog-log and probit built-in link functions to periodontal disease ordinal data.

**Table 2:** Performances ordinal regression model with different built-in link functions

Ordinal model with	Log likelihood	Akaike information criteria
Logit link function	−2078.36	2.39
Probit link function	−2099.90	2.41
Clog-log link function	−1908.49	2.19
nlog-log link function	−1992.05	2.29

## DISCUSSIONS AND CONCLUSIONS

It is convenient for us to analyze ordinal outcome by means of logistic and linear regression analyses. By altering the measuring scale of ordinal outcome, we are able to analyze data and produce research findings. However, the loss of information or incorrect analysis may have occurred in some cases. For instance, when the scale of outcome categories (e.g., healthy, bleeding calculus; shallow pocket and deep pocket) is arbitrarily collapsed into a binary measure (e.g., without disease and with disease), we are forced to use logistic regression analysis to analyze the two levels of ordinal outcome. By doing so, important information may be lost in the resulting model. Therefore, we study the effects of independent variables on all levels of the ordered categorical outcome; an ordinal regression method must be appropriately chosen in order to obtain valid research results. Using the ordinal regression method, researchers could identify significant independent variables with their control to enhance occurrence of periodontal disease.

We agree with Ananth and Kleinbaum[16]; Scott, Goldberg and Mayo[18]; Rolf and Axel[26] that ordinal regression models should be more widely used in epidemiology and biomedical research, especially in dental epidemiology. However, for adequate use, one has to be very careful about the goodness of fit and validity of model assumptions. If the usual assumption of equal slopes for all ordinal response levels is fulfilled by the data, the standard models with different built-in link functions (logit, probit, Clog-log, nlog-log represent the powerful tools producing easily interpretable parameters which summarize the effects of independent variables over all response levels. In the case of ordinal responses, much more effort by the researcher themselves is required to find models describing the data adequately. Nowadays different statistical softwares offer an easy access to the standard ordinal regression models with built-in link functions (logit, probit, Clog-log, n (n)log-log).[19,27]

On analyzing the results of this study, negligible differences were observed in ordinal models with different built in link functions with their log likelihood estimates and comparable in practical applications of periodontal disease data. This can be explained by the fact that the ordinal regression models with different built-in link functions are equivalent in any case.[28] On the other hand, all the link functions are quite similar, at least for small probabilities.[9] Then again, all built-in link functions would usually not lead to quite different estimated associations between the independent variables and the response variable. All built-in link functions that were considered here did not result in quite different estimates of response, but found differences in likelihood ratio chi-square values. The ‘goodness of fit’ statistic was acceptable, but similar to Pearson’s and deviance methods.

In summary, there are no differences of practical relevance in ordinal responses of periodontal disease between the results of models with four built-in link functions. All built-in link functions provided similar findings, which must be checked carefully before a model with link can be applied adequately. The choice of the model with built-in link functions depends on the researcher’s preference.[29]
